# Robust Non-Invasive Cardiac Index Prediction via Feature Integration and Data-Augmented Neural Networks

**DOI:** 10.3390/bioengineering13040477

**Published:** 2026-04-18

**Authors:** Chih-Hao Chang, Mei-Ling Chan, Yu-Hung Fang, Po-Lin Huang, Tsung-Yi Chen, Tsun-Kuang Chi, I Elizabeth Cha, Tzong-Rong Ger, Kuo-Chen Li, Shih-Lun Chen, Liang-Hung Wang, Jia-Ching Wang, Patricia Angela R. Abu

**Affiliations:** 1 Department of Computer Science and Information Engineering, National Central University, Taoyuan 320317, Taiwan; 113582001@cc.ncu.edu.tw (C.-H.C.); jcw@csie.ncu.edu.tw (J.-C.W.); 2 School of Physical Educational College, Jiaying University, Meizhou 514000, China; lynn202207017@jyu.edu.cn; 3 Department of Pulmonary and Critical Care Medicine, Chiayi Chang-Gung Memorial Hospital, Chang-Gung Medical Foundation, Chiayi 613016, Taiwan; 8902062@gmail.com; 4 Department of Electronic Engineering, Feng Chia University, Taichung 407102, Taiwan; m1317207@o365.fcu.edu.tw (P.-L.H.); tsungychen@fcu.edu.tw (T.-Y.C.); 5 Department of Electrical Engineering, Ming Chi University of Technology, New Taipei City 243303, Taiwan; simonchi@mail.mcut.edu.tw; 6 Undergraduate Program in Intelligent Computing and Big Data, Chung Yuan Christian University, Taoyuan City 320314, Taiwan; 7 Department of Biomedical Engineering, National Yang Ming Chiao Tung University, Taipei 112304, Taiwan; sunbow@nycu.edu.tw; 8 Department of Information Management, Chung Yuan Christian University, Taoyuan City 320314, Taiwan; kuochen@cycu.edu.tw; 9 Department of Electronic Engineering, Chung Yuan Christian University, Taoyuan 320314, Taiwan; 10 Research Center for Semiconductor Materials and Advanced Optics, Chung Yuan Christian University, Taoyuan City 320314, Taiwan; 11 Department of Microelectronics, College of Physics and Information Engineering, Fuzhou University, Fuzhou 350108, China; eetommy@mail2000.com.tw; 12 Department of Information Systems and Computer Science, Ateneo de Manila University, Quezon City 1108, Philippines; pabu@ateneo.edu

**Keywords:** cardiac index, non-invasive sensing, IoT, IoMT, artificial neural networks, machine learning, physiological signal processing, data augmentation, cardiovascular disease assessment

## Abstract

Concurrent with the rising consumption of ultra-processed, high-calorie diets and the decline in physical activity, obesity and related cardiovascular conditions among young adults have continued to increase, becoming an important global public health concern. This study integrates non-invasive Internet of Things (IoT) sensing devices, including the TERUMO ES-P2000 blood pressure monitor (Terumo Corp., Tokyo, Japan) and the PhysioFlow PF07 Enduro cardiac hemodynamic analyzer (Manatec Biomedical, Poissy, France), with an artificial neural network (ANN) for cardiac index (CI) prediction. Through appropriate data preprocessing and model training strategies, the generalization ability and stability of the proposed CI prediction model were significantly enhanced. Experimental results demonstrate that, when using three physiological parameters as input, the ANN achieved a classification accuracy of 97.78%, substantially outperforming traditional approaches. Even under two-parameter input conditions, the model maintained strong predictive performance. These findings confirm the effectiveness and practical potential of the proposed framework for real-time, non-invasive CI assessment. Moreover, this research has received rigorous assessment and approval from the Institutional Review Board (IRB) under application number 202501987B0.

## 1. Introduction

Cardiovascular disease (CVD) has become a major global health burden, with its onset age steadily decreasing, particularly among younger populations aged 20 to 29. Reports from the World Health Organization indicate that metabolic conditions such as obesity, hypertension, hyperlipidemia, and diabetes are increasingly prevalent in younger age groups, thereby accelerating the early onset of CVD [1]. Furthermore, previous studies have shown that obesity-related indicators, including body mass index and waist circumference, are significantly associated with the incidence of atrial fibrillation and ischemic stroke, highlighting the critical importance of early cardiovascular risk assessment in preventive medicine [2].

In clinical practice, the Cardiac Index (CI) is a key indicator reflecting cardiac output normalized to body surface area and is widely used for cardiovascular function assessment and disease stratification. However, conventional CI measurement relies heavily on specialized hemodynamic analyzers such as PhysioFlow, which require controlled clinical environments and expert operation. These constraints hinder the clinical application of CI monitoring in routine screening, long-term surveillance, and home-based healthcare scenarios. Therefore, developing an accurate and stable CI prediction method using minimal, non-invasive parameters remains a paramount research challenge. With the rapid advancement of the Internet of Things (IoT) and Internet of Medical Things (IoMT) technologies, non-invasive sensing devices are increasingly capable of continuously acquiring multiple physiological signals in real time, while leveraging cloud and edge computing architectures for efficient data integration and analysis [3,4]. When combined with machine learning techniques, these systems enable automated decision-making and significantly enhance the efficiency of early disease detection and health management [5,6,7,8,9,10].

Among various approaches, Artificial Neural Networks (ANNs) have demonstrated strong capabilities in modeling complex nonlinear physiological relationships and have consistently outperformed traditional statistical methods in cardiovascular prediction tasks [11,12,13,14,15,16]. Recent studies have further explored the integration of IoT sensing and artificial intelligence for cardiovascular risk monitoring and multi-disease prediction. For instance, IoT-based AI frameworks have been proposed for monitoring cardiovascular risk in young obese populations [17], while data-driven predictive architectures and deep auto-optimized collaborative learning (DACL) approaches have shown superior performance in multi-disease prognosis [18,19]. In addition, ANN-based methods have successfully estimated critical hemodynamic parameters, such as cardiac output, directly from physiological signals, demonstrating the feasibility of non-invasive prediction [20]. Furthermore, IoT-enabled smart healthcare systems have been widely applied in the reliable diagnosis of metabolic diseases, such as diabetes [21]. However, most existing studies rely on high-dimensional input features or comprehensive clinical datasets, which limits their applicability in real-world scenarios. When applied to practical environments with limited and non-invasive inputs, these methods often suffer from reduced prediction accuracy and decreased model stability. Moreover, traditional multiple linear regression approaches fail to capture nonlinear physiological interactions, resulting in predictions biased toward mean values. In addition, insufficient data preprocessing further degrades overall model reliability.

Motivated by these limitations, this study focuses on developing a non-invasive CI prediction method that is both accurate and practically deployable under limited physiological input conditions. We propose a framework that integrates IoT-based sensing with an optimized Feedforward Neural Network (FFNN), incorporating systematic data preprocessing and Mixup-based data augmentation to enhance nonlinear feature learning and generalization capability. Compared with existing approaches, the proposed method maintains high predictive performance while reducing the number of required input parameters, thereby improving its practicality and efficiency for real-world applications. Furthermore, a dual-task evaluation framework is adopted to explicitly distinguish between binary CI classification (CI ≥ 3 vs. CI < 3) [22,23] and continuous CI regression prediction, avoiding ambiguity in performance interpretation and enhancing result interpretability. To validate the stability and reliability of the proposed method, an independent test set consisting of original clinical samples was reserved, and cross-validation was employed for comprehensive evaluation. Experimental results demonstrate that, even under limited sample conditions, the proposed model achieves consistent and robust predictive performance. In particular, the model exhibits high accuracy in classification tasks, indicating strong potential for real-world deployment in non-invasive cardiovascular monitoring and intelligent healthcare systems.

## 2. Methodology

**Figure 1:**
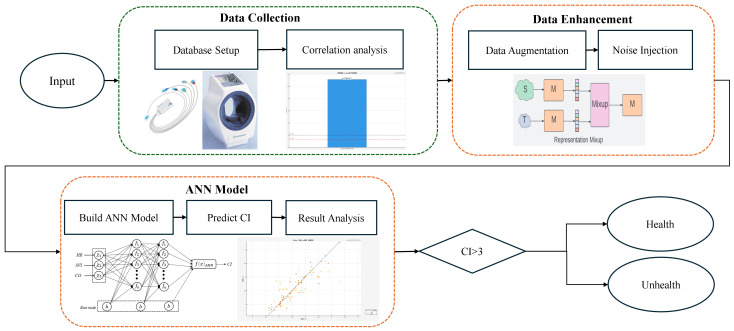
The flowchart used in this study.

This study integrates IoT and IoMT sensing technologies with machine learning techniques to monitor cardiovascular function and predict CI in young adults with obesity. The research process is divided into several stages, as illustrated in Figure 1. First, physiological data were acquired using advanced non-invasive sensing devices, including TERUMO ES-P2000 (Terumo Corp., Tokyo, Japan), InBody 720,(InBody Co., Ltd., Seoul, South Korea), and PhysioFlow (Manatec Biomedical, Poissy, France), to establish the input features for subsequent analysis. Next, the collected health indicators are standardized to eliminate the effects of differing units on the model. Subsequently, data augmentation is applied to diversify the training dataset and enhance the model’s generalization capability. Finally, an ANN model is developed and evaluated to predict CI.

### 2.1. Data Collection

**Figure 2:**
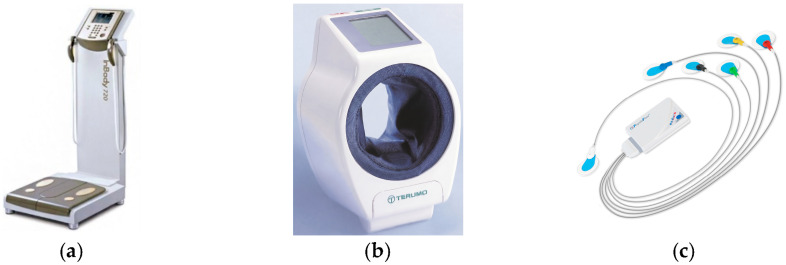
Sensing instrument used in this study. (**a**) InBody 720 body composition analyzer. (**b**) TERUMO ES-P2000 blood pressure monitor. (**c**) PhysioFlow blood flow analyzer.

**Figure 3:**
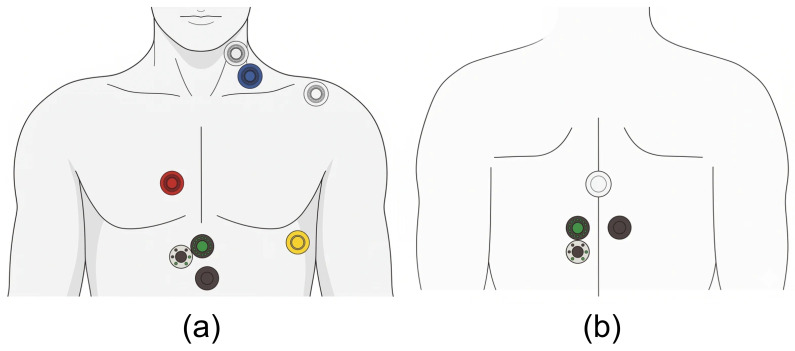
(**a**) Paste position. (**b**) Sensor stickers on the back of the body.

This study enrolled 54 obese individuals who were between 20 and 29 years old and had a body mass index (BMI) exceeding 27 kg/m^2^. All participants were free of diagnosed cardiovascular disease and the Taiwan Ministry of Health and Welfare body composition guidelines served as the basis for obesity determination. Data collection was performed using three specialized non-invasive instruments. Hemodynamic parameters, including blood pressure (SBP, DBP, PP, MAP) and heart rate (HR), were recorded using the TERUMO ES-P2000. Cardiac function, specifically stroke volume (SV), was assessed via the PhysioFlow analyzer, while the InBody 720 was employed to determine body composition, including BMI and fat distribution. Blood flow and cardiac hemodynamic measurements were conducted after sensor placement and monitoring procedures, which are depicted in Figure 2 and Figure 3.

The experimental protocol was conducted in sequential stages. Initially, participants rested in a seated position for 10 min to ensure physiological stabilization, followed by a 3 min blood pressure assessment. Subsequently, electrodes were attached for a 10 min recording of cardiac physiological signals, after which a further 5 min rest period was observed. The protocol concluded with a 20 min assessment of cardiac hemodynamic parameters. The complete sequence and temporal allocation are summarized in Table 1.

In this study, sample labeling for cardiovascular status was based on a clinically established cardiac index (CI) threshold. This threshold is well supported by authoritative cardiovascular physiology literature and was further validated through expert consensus involving three clinicians with more than three years of clinical experience, and a physiology specialist. Specifically, CI ≥ 3 L/min/m^2^ was defined as normal, while CI < 3 L/min/m^2^ was categorized as abnormal [22,23]. All labels were objectively determined from CI values directly measured using the PhysioFlow hemodynamic analyzer, ensuring consistency, reproducibility, and minimizing subjective bias.

### 2.2. Data Analysis

This study employed statistical analyses to examine associations among obesity-related measures and cardiac-function parameters. The study employed descriptive statistics to present demographic information and health data of 54 participants while analyzing essential cardiovascular measurements, including body weight, BMI, blood pressure, and HR. The initial summary of subject health status helped establish basic data that would support further detailed investigations. The Pearson product-moment correlation analysis [24] tested how body weight related to cardiac hemodynamic parameters including SVI, CO, and EF to establish whether body weight impacts cardiac function. The analysis used SPSS version 24.0 with statistical significance determined by *p* < 0.05 for drawing reliable conclusions. The research investigated how different physiological indicators connect with CI along with body weight correlations to SV and HR while stressing early cardiovascular risk assessment in individuals with obesity.

### 2.3. Data Enhancement

This study aims to improve predictive accuracy for the cardiac index (CI). Traditional CI assessment has primarily relied on physiological indicators such as HR and SVI; however, these indicators have limitations in accurately reflecting cardiac function. To address these limitations, systematic data preprocessing and augmentation strategies were integrated to enhance model robustness and predictive performance. The dataset consisted of measurements collected from 54 subjects, resulting in a total of 270 samples. These samples were divided into training and testing sets in an 8:2 ratio, yielding 216 training samples and 54 testing samples. In addition, 30% of the training set was further reserved as a validation set for model tuning and early stopping.

To improve the model’s generalization ability and robustness, the training data were augmented sixfold using Mixup [25] and Noise Injection [26]. Mixup was applied by randomly combining two training samples through a linear combination to generate new synthetic samples, as illustrated in Equations (1) and (2). To further enhance generalization and increase training data diversity, zero-mean Gaussian noise with standard deviation σ was added to each feature vector after Mixup, introducing slight random perturbations around the original feature space, as described in Equation (3). Consequently, the training set expanded from 216 samples to 1296 samples after augmentation.

(1) $$\tilde{x}=\lambda x_{i}+\left(1-\lambda \right)x_{j}$$

(2) $$\tilde{y}=\lambda y_{i}+(1-\lambda )y_{j}$$

(3) $$\hat{x}=\tilde{x}+\epsilon ,\epsilon ∼N(0,\sigma^{2})$$

It is important to note that the augmented data included both original and synthetic samples and did not represent independent subjects. The test set remained completely independent and was not subjected to any augmentation or model training processes.

To evaluate the impact of different variable combinations on predictive performance, multiple prediction models were developed, including simplified models based on HR, cardiac output (CO), and SVI, as well as multivariate models incorporating multiple physiological parameters. This comparative approach helps identify the most efficient set of input features without compromising prediction accuracy, thereby simplifying the measurement process and improving clinical applicability.

### 2.4. ANN Model

**Figure 4:**
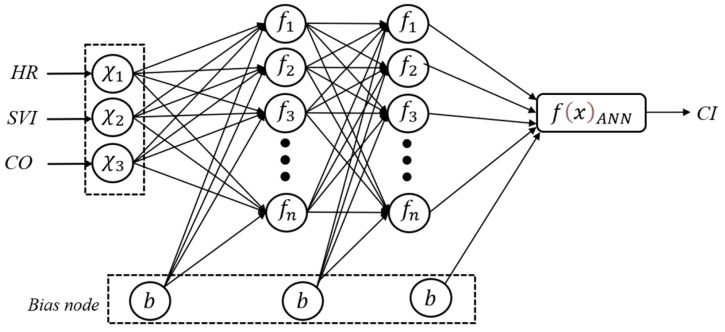
Schematic of the FFNN architecture.

An Artificial Neural Network (ANN) was utilized as the primary predictive architecture for this study. Leveraging their capacity for high-dimensional nonlinear modeling, ANNs are particularly effective at capturing the complex, interdependent relationships between physiological variables and cardiac output—tasks where traditional linear regressions often fall short [27]. In the medical domain, ANNs are highly suitable for physiological parameter prediction and health risk assessment, as they can learn and represent complex physiological variations through multilayer neural structures [28]. The ANN architecture comprises an input layer for physiological features (e.g., HR, CO), hidden layers for nonlinear feature transformation, and an output layer for CI prediction. Weighted summations and activation functions within the hidden layers enable the model to learn complex relationships between inputs. The overall ANN architecture is illustrated in Figure 4.

A Feedforward Neural Network (FFNN) was utilized as the core predictive model, implementing a backpropagation learning rule to minimize residual errors and ensure robust performance. The hidden layers employ the Rectified Linear Unit (ReLU) activation function to improve nonlinear feature learning, while the output layer uses a linear activation function to accommodate the regression-based prediction of CI. The proposed FFNN architecture comprises three hidden layers with 32, 16, and 8 neurons, respectively, with each layer fully connected. The input features are transformed through weight and bias adjustments and passed through the ReLU activation function for nonlinear computation, as defined in Equation (4), while the linear combination for the output layer is represented in Equation (5). To prevent overfitting, a 30% dropout rate was applied after the first and second hidden layers [29], randomly deactivating a subset of neurons during training to encourage the model to learn more generalizable feature representations. After multiple training iterations, the FFNN automatically adjusted the connection weights and biases to achieve optimal predictive performance. The final model demonstrated high stability and precision in CI prediction under various physiological conditions, exhibiting strong generalization capability and significant potential for clinical application in cardiovascular health monitoring.

(4) $$h_{j}^{\left(l\right)}=\phi \left(\sum_{i=1}^{n^{\left(l-1\right)}}w_{ij}^{\left(l\right)}h_{i}^{\left(l-1\right)}+b_{j}^{\left(l\right)}\right)$$

(5) $$\hat{y}=\sum_{j=1}^{n^{\left(L\right)}}w_{j}^{\left(out\right)}h_{j}^{\left(L\right)}+b^{\left(out\right)}$$

To comprehensively evaluate model performance, this study adopted a dual-task evaluation framework that explicitly distinguished between classification and regression tasks. For the classification task (CI ≥ 3 vs. CI < 3), the model performance was evaluated using standard classification metrics, including Accuracy, Recall (Sensitivity), Specificity, Precision, and F1-score. These metrics provide a comprehensive assessment of the model’s ability to correctly identify normal and abnormal cardiac function. For the regression task (continuous CI prediction), model performance is evaluated using both statistical error metrics and confidence-based accuracy. Specifically, the proportion of predictions falling within the 95% confidence interval of the actual values is defined as Accuracy, reflecting the degree to which model predictions align with the uncertainty range of the true data. Furthermore, the Coefficient of Determination (R^2^), Root Mean Square Error (RMSE), Mean Absolute Error (MAE), and Mean Absolute Percentage Error (MAPE) are employed, as defined in Equations (6)–(9). R^2^ measures the proportion of variance in the target variable that can be explained by the model, with values approaching 1 indicating a better fit. RMSE quantifies the standard deviation of prediction errors and is particularly sensitive to large deviations, reflecting overall prediction stability. MAE represents the average absolute difference between predicted and actual values, providing a direct measure of prediction error magnitude. MAPE further normalizes prediction error in percentage form, enabling intuitive interpretation in clinical contexts. This dual-task evaluation framework ensures a clear separation between classification accuracy and regression accuracy, thereby avoiding ambiguity and providing a more rigorous and interpretable assessment of model performance.

(6) $$\mathrm{Accuracy}=\frac{\mathrm{Number}\;\mathrm{of}\;\mathrm{predictions}\;\mathrm{within}\;95\%\;\mathrm{confidence}\;\mathrm{interval}}{\mathrm{Total}\;\mathrm{number}\;\mathrm{of}\;\mathrm{predictions}}\times 100\%$$

(7) $$R^{2}=1-\frac{\sum_{i=1}^{n}({\mathrm{Actual}\;\mathrm{Value}}_{i}-{\mathrm{Predicted}\;\mathrm{Value}}_{i}{)}^{2}}{\sum_{i=1}^{n}({\mathrm{Actual}\;\mathrm{Value}}_{i}-\bar{\mathrm{Actual}\;\mathrm{Values}}{)}^{2}}$$

(8) $$RMSE=\sqrt{\frac{1}{n}\sum_{i=1}^{n}{\left({\mathrm{Predicted}\;\mathrm{Value}}_{i}-{\mathrm{Actual}\;\mathrm{Value}}_{i}\right)}^{2}}$$

(9) $$MAE=\frac{1}{n}\sum_{i=1}^{n}|{\hat{y}}_{i}-y_{i}|$$

### 2.5. Model Configuration and Implementation Details

The ANN model was configured using the hyperparameters listed in Table 2. The dataset was divided into a training set (80%, 216 samples) and an independent testing set (20%, 54 samples). Within the training partition, the samples were further subdivided: 70% were used as the training subset for weight optimization, whereas 30% were allocated as the validation subset for early stopping and hyperparameter tuning. The testing set was reserved exclusively for final performance evaluation. Input features were normalized using z-score normalization, and the model was trained using the Levenberg–Marquardt optimization algorithm. Training was implemented via the MATLAB trainlm function, utilizing full-batch optimization (batch size *n* = 216). Rather than a fixed learning rate, the Levenberg–Marquardt algorithm employs an adaptive damping factor (*μ*) to ensure convergence, with an initial *μ* of 0.001 and adjustment factors of 0.1 (decrease) and 10 (increase). All experiments were conducted on the hardware and software platform summarized in Table 3. The training process was performed on a workstation equipped with an Intel Core Ultra 7 265K CPU (Intel Corp., Santa Clara, CA, USA), 32 GB RAM, and an NVIDIA GeForce RTX 4080 GPU (NVIDIA Corp., Santa Clara, CA, USA), running on Windows 11 with MATLAB R2025b.

## 3. Result

This study aims to integrate IoT and IoMT technologies to collect multiple physiological parameters from subjects in real-time using non-invasive sensors. To enhance the model’s adaptability to data diversity and mitigate the limitation of insufficient data volume, data augmentation techniques were applied to improve model generalization and stability. The collected and augmented data were then used to train an FFNN model, establishing a highly accurate and interpretable CI prediction system. This approach effectively enhanced the precision and robustness of CI prediction, thereby enhancing the model’s clinical applicability. Furthermore, the proposed system demonstrates the broad potential of combining IoT technologies with advanced machine learning methods to enable intelligent cardiovascular health monitoring and risk prediction. Future work may further integrate multimodal physiological data and extend toward long-term dynamic monitoring to strengthen personalized cardiovascular health management.

The findings of this study are presented in two major components. The first part analyzes physiological parameters using statistical methods to examine the relationships between various physiological indicators and cardiac functional indices, thereby identifying key factors that influence cardiac performance. The second part evaluates the performance of ANN models by comparing different network architectures and parameter scales on the same dataset, assessing their accuracy and multiple performance metrics in predicting CI. Through this two-level analysis, the study not only provides a comprehensive understanding of the associations between physiological characteristics and cardiac health but also verifies the feasibility and robustness of the FFNN model in predicting cardiac function and overall physiological status. These results offer valuable insights for future clinical applications and the early management of cardiovascular disease risk.

### 3.1. Results of Statistical Analysis of Physiological Parameters

Table 4 presents the physical relationships among physiological parameters, while Table 5 illustrates the statistically significant correlations among cardiac hemodynamic parameters. The results indicate that body weight has a notable impact on cardiac function. As one of the key indicators of cardiac performance, CI shows a strong positive correlation with both the SVI (*r* = 0.521, *p* < 0.01) and CO (*r* = 0.914, *p* < 0.01), suggesting that higher CI values are typically associated with greater cardiac pumping efficiency. In contrast, CI exhibits a significant negative correlation with End-Systolic Volume (ESV) (*r* = −0.445, *p* < 0.01), implying that enhanced myocardial contractility results in a smaller residual blood volume within the ventricle at the end of systole, thereby reflecting improved ventricular contraction performance.

Overall, these findings highlight the importance of CI as a critical indicator for assessing cardiac function and demonstrate its clinical applicability in evaluating cardiovascular health. Furthermore, the results emphasize the importance of continuous monitoring of cardiovascular parameters, particularly in cases where increased body weight contributes to a higher risk of hypertension and heart disease. Such monitoring supports effective clinical management and early prevention of cardiovascular complications.

### 3.2. FFNN Model Training

**Figure 5:**
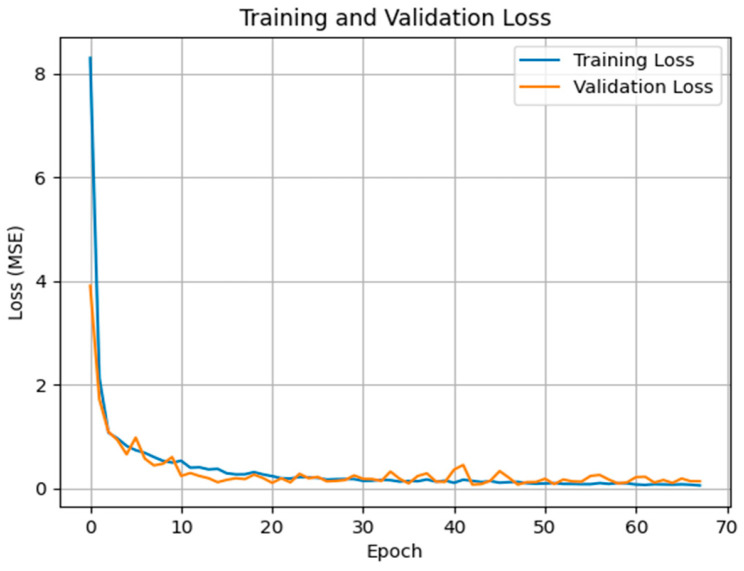
Training and Validation Loss Curve.

Data were partitioned into training and validation sets to refine the FFNN’s predictive accuracy. This approach allowed for continuous monitoring of model performance, ensuring high generalizability and preventing overfitting. As illustrated in Figure 5, the training process incorporated an Early Stopping mechanism to monitor both training and validation losses [30], thereby evaluating the model’s learning behavior and generalization capability. The training loss exhibited a sharp decline at the beginning, decreasing from approximately 8.3 to nearly 0.1, indicating that the model effectively captured key features and continuously optimized its parameters. The validation loss also showed a significant initial decrease and remained stable around 0.15 throughout the training, demonstrating the absence of overfitting and consistent generalization performance. Both training and validation loss curves flattened between epochs 40 and 50, signaling convergence. Training automatically terminated at epoch 68 according to the predefined Early Stopping criterion, which halted the process after the validation loss failed to improve for 20 consecutive epochs. This approach ensured that the optimal weight configuration was selected for maximum generalization and stable predictive performance.

The overall loss trajectory indicates that the FFNN achieved efficient and stable learning, effectively minimizing prediction errors. This outcome establishes a reliable foundation for accurate CI prediction and related physiological parameter estimation.

### 3.3. FFNN Model Results

To avoid ambiguity arising from combining classification and regression metrics under a single “Accuracy” definition, this study reports model performance using two distinct evaluation frameworks, as described in Section 2.4.

(a) Classification Performance

Table 6 presents the classification performance of the proposed FFNN and three representative machine learning models—Support Vector Machine (SVM), Support Vector Regression (SVR), and K-Nearest Neighbors (KNN)—evaluated on a held-out test set consisting of 54 original clinical samples. The FFNN achieved high classification performance, with an accuracy of 97.78% on the test set, correctly identifying the majority of samples with only minimal misclassification. This result indicates that the model is highly effective in distinguishing between normal (CI ≥ 3) and abnormal (CI < 3) cardiac function. In comparison, SVR and SVM also demonstrated strong classification performance, both achieving an accuracy of 95.82%, while KNN achieved 93.87%. Although these conventional machine learning models exhibit competitive performance, the FFNN consistently outperformed them in terms of accuracy and stability, highlighting its superior capability in modeling nonlinear physiological relationships.

Furthermore, to evaluate the stability and generalization capability of the proposed model, a 5-fold cross-validation (5-fold CV) was conducted. As shown in Table 7, the FFNN exhibited consistent and stable performance across different data partitions, with only minor variations observed among folds. This indicates that the proposed model is not sensitive to specific data splits and is capable of maintaining reliable learning performance across different training subsets. These results further suggest that the superior classification performance of the FFNN is not attributed to overfitting or favorable data partitioning, but rather stems from its effective ability to capture the underlying nonlinear relationships among physiological parameters.

(b) Regression Performance

Table 8 presents the regression performance for continuous CI prediction, evaluated using the proportion of predictions falling within the 95% confidence interval of the actual CI values, alongside R^2^, RMSE, MAE, and MAPE. The FFNN achieved an R^2^ of 0.929, RMSE of 0.1670, MAE of 0.1236, and MAPE of 3.12%, outperforming all comparison models including SVR, Random Forest, KNN, and MLR. These results indicate that the predicted CI closely approximates the measured CI, confirming the clinical viability of the proposed non-invasive approach. It is important to note that the “Accuracy” in this table refers specifically to the proportion of predictions within the 95% confidence interval of the actual values, and is therefore not directly comparable to the classification accuracy reported in Table 6.

**Figure 6:**
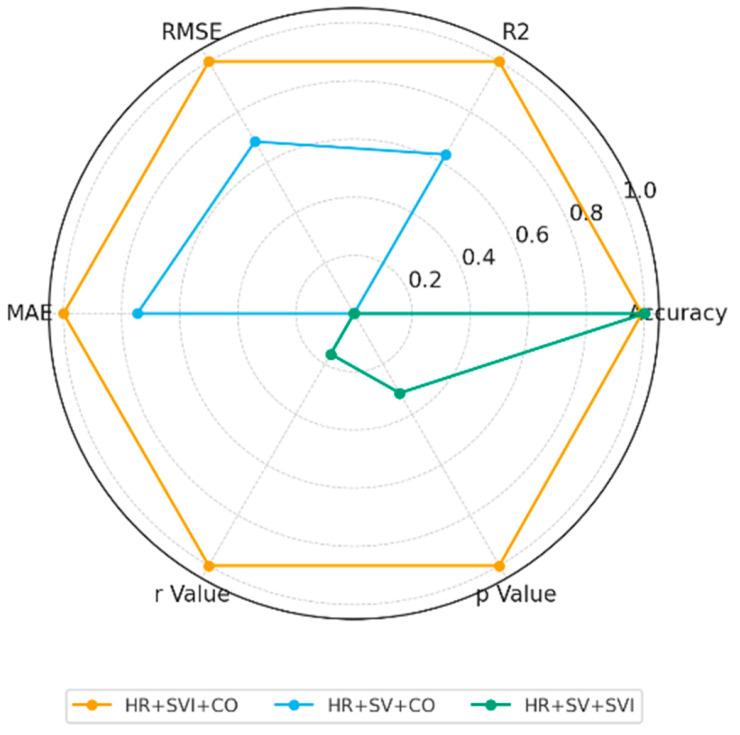
Normalized Radar of HR/SV/SVI/CO Input Combinations.

Building upon these findings, the study further examined how different combinations of cardiovascular indicators influence CI prediction performance to evaluate the model’s robustness under varying input conditions. As shown in Table 9 and Figure 6, the combination of SVI, HR, and CO yielded the best overall performance, achieving an MAE of 0.1236, an R^2^ of 0.929, and attaining high classification performance on the held-out test set. It should be noted that this classification accuracy specifically refers to the binary classification task (CI ≥ 3 vs. CI < 3) and should not be confused with the regression-based accuracy defined under the 95% confidence interval. These results demonstrate that the three-to-one configuration (HR + SVI + CO) most effectively captures cardiac functional variations and provides stable, high-accuracy predictions.

Furthermore, to assess the model’s scalability when fewer physiological indicators are available, the study evaluated several two-to-one input combinations (HR + SV, HR + SVI), with results summarized in Table 10. Despite reduced data dimensionality, the model maintained stable predictive performance. Among the evaluated combinations, HR + SVI achieved the best balance (MAE = 0.350, R^2^ = 0.442), indicating that the model can still effectively capture cardiac hemodynamic patterns even with limited inputs.

Overall, the ANN architecture demonstrated strong flexibility and generalization capability. The three-input configuration achieved the best predictive performance and clinical relevance, while the two-input configurations showed promising potential for maintaining reliable predictions when fewer physiological inputs are available.

These findings suggest that the model’s ability to integrate varying physiological inputs provides a robust and adaptable solution for real-world applications. By maintaining predictive stability even in reduced-input scenarios, the proposed framework offers a practical approach to simplifying data acquisition while preserving accuracy, thereby enhancing its applicability for real-time, non-invasive CI prediction.

## 4. Discussion

This study successfully integrated IoT and IoMT technologies to collect multiple physiological parameters through non-invasive sensing and employed data augmentation and AI algorithms to enhance the accuracy and stability of CI prediction. The proposed FFNN demonstrated outstanding predictive performance, achieving a classification accuracy of 97.78%, a coefficient of determination of 0.929, and a mean absolute error of 0.1236. Compared with conventional multiple linear regression models that achieved only 53.33% accuracy, the proposed network exhibited superior nonlinear learning and adaptive optimization capabilities, effectively capturing the complex hemodynamic relationships within physiological data. These results confirm the high reliability and clinical applicability of the proposed model.

The analysis of different input feature combinations revealed that the model performed best when HR, SVI, and CO were used as input variables. Even when the input dimension was reduced to two features, such as HR with CO or SVI, the network maintained strong predictive stability and regression performance. This demonstrates that the neural architecture remains robust and generalizable even when fewer sensing parameters are available, highlighting its potential for practical deployment in clinical or portable healthcare environments where sensor availability may be limited.

The results further indicate that the model can effectively predict CI variations across diverse subjects, including extreme values, even when using a limited number of physiological indicators. This capability suggests that the proposed approach is not only suitable for general cardiovascular monitoring but also for early-stage assessment in high-risk or abnormal cases. Building upon these promising outcomes, future work will focus on optimizing the model to maintain high classification accuracy while improving the precision of CI estimation using a minimal set of input features, thereby expanding the versatility and applicability of the system.

Nevertheless, several limitations should be acknowledged. First, the study is based on a relatively small sample size, which may limit the generalizability of the findings. Second, although data augmentation techniques were employed to enhance model robustness, augmented data do not represent independent subjects. Therefore, future work will focus on validating the proposed framework using larger and more diverse populations to further improve its generalization capability and clinical reliability.

## 5. Conclusions

In conclusion, this study demonstrated that integrating IoT- and IoMT-based sensing technologies with an FFNN significantly improves the accuracy, robustness, and clinical value of non-invasive CI prediction. The proposed model achieved 97.78% accuracy, a coefficient of determination of 0.929, and a mean absolute error of 0.1236, outperforming traditional regression methods. The ability to maintain strong predictive performance using only a small number of physiological features highlights its scalability, adaptability, and suitability for real-world clinical environments.

Future work will aim to refine the network to achieve accurate prediction across all subjects, including outlier cases, using minimal sensing parameters. This improvement will broaden the system’s applicability and facilitate its integration into intelligent, low-cost, and real-time cardiovascular health monitoring platforms. Expanding the dataset to include more diverse populations and incorporating multimodal signals with temporal deep learning models such as LSTM and GRU will further enhance the capability for dynamic cardiac trend analysis and personalized healthcare.

## Figures and Tables

**Table 1:** Blood Pressure, Cardiac Hemodynamic Assessment Process.

10 min	3 min	10 min	5 min	20 min
Sit and rest	Blood pressure	Paste electrodes	Sit and rest	Detection of cardiac hemodynamic parameters

**Table 2:** ANN Hyperparameter Configuration.

Parameter	Value
Architecture	(32, 16, 8)
Training epochs	100
Batch Size	Full-batch (216 samples)
Initial Damping Factor (*μ*)	0.001
*μ* decrease/increase factor	0.1/10
Normalization method	Z-score standardization
Optimizer	Levenberg–Marquardt

**Table 3:** Hardware and Software Configuration.

Platform	Component	Specification
Hardware	CPU	Intel Core Ultra 7 265K
	GPU	NVIDIA GeForce RTX 4080
	RAM	32 GB
Software	Operating System	Windows 11
	Environment	Matlab 2025b

**Table 4:** Relationships Among Physiological Parameters.

Parameter	Description	Formula	Reference Value
Stroke Volume (SV) (mL)	The volume of blood ejected by the heart per beat.	SV = EDV − ESV	70 mL
Stroke volume index (SVI) (mL/m^2^)	Stroke volume per body surface area.	SVI = SV/m^2^	40–50 mL/m^2^
Cardiac Output (CO) (L/min)	The amount of blood ejected by the left ventricle per minute.	CO = SV × HR	4.0–6.5 L/min
Cardiac Index (CI) (L/min/m^2^)	Cardiac output per minute per body surface area.	CI = CO/m^2^	3 L/min/m^2^
End Diastolic Volume (EDV) (mL)	The volume of blood when the left ventricles are filled with oxygenated blood.	EDV = SV + ESV	108 + 24 mL
End Systolic Volume (ESV) (mL)	The amount of blood remaining in the ventricle after ejection.	ESV = EDV − SV	45 + 46 mL
Ejection Fraction % (EF%)	The ratio of the volume of blood ejected from each ventricle.	EF% = (SV/EDV) × 100	65–70%

**Table 5:** Correlation coefficient matrix between body weight and Cardiac Hemodynamic parameters—r value (n = 54).

	Body Weight	SV	SVI	CO	CI	VET	EDV	ESV	EF%
Body Weight	1	0.234	−0.310 *	0.238	−0.153	0.034	0.381 **	0.350 **	−0.213
SV		1	0.837 **	0.529 **	0.445 **	−0.022	0.601 **	0.103	0.353 **
SVI			1	0.371 **	0.521 **	−0.035	0.361 **	−0.097	0.458 **
CO				1	0.914 **	−0.204	0.163	−0.292 *	0.593 **
CI					1	−0.214	0.002	−0.445 **	0.694 **
VET						1	0.177	0.205	−0.132
EDV							1	0.796 **	−0.395 **
ESV								1	−0.858 **
EF%									1

* p < 0.05; ** p < 0.1.

**Table 6:** Model Comparison for CI Classification (CI ≥ 3 vs. CI < 3).

Metric	FFNN	SVR [30]	SVM [31]	KNN [32]
Accuracy	97.78%	95.82%	95.82%	93.87%
Recall	97.78%	95.56%	97.78%	95.56%
Specificity	97.78%	97.78%	80.00%	80.00%
Precision	97.78%	97.78%	95.64%	95.56%
F1-score	97.78%	96.70%	96.75%	95.56%

**Table 7:** 5-Fold Cross-Validation Results of FFNN Classification Accuracy.

	Fold1	Fold2	Fold3	Fold4	Fold5	Average
5-Fold CV Accuracy	96.30%	100.00%	94.44%	98.15%	100.00%	**97.78%**

**Table 8:** Regression Metrics for Continuous CI Prediction.

Metric	FFNN	SVR [30]	Random Forest [33]	KNN [32]	MLR [17]
Accuracy	**96.00%**	96.00%	96.00%	96.00%	92.00%
R^2^	**0.929**	0.8988	0.9019	0.7788	0.6767
RMSE	**0.1670**	0.2060	0.2281	0.2823	0.3541
MAE	**0.1236**	0.1395	0.1739	0.2274	0.2725
MAPE	**3.12%**	3.36%	4.84%	6.4%	5.55%
r Value	**0.9368**	0.9481	0.9497	0.8924	0.8226
*p* Value	**0.0000**	0.0000	0.0000	0.0000	0.0000

**Table 9:** Cardiac Index Prediction Performance Using Three Physiological Input Parameters.

	HR + Any 2 Parameters			
	HR + SVI + CO	HR + SV + CO	HR + SV + SVI	Average
Accuracy	**97.78%**	90%	97.78%	95.19%
R^2^	**0.929**	0.595	0.249	0.591
RMSE	**0.167**	0.341	0.599	0.369
MAE	**0.1236**	0.257	0.501	0.2939
r Value	**0.9368**	0.7816	0.8013	0.8399
*p* Value	**0.0000**	0.0076	0.0053	0.0043

**Table 10:** Cardiac Index Prediction Performance Using Two Physiological Input Parameters (Reduced Input Condition).

	HR + Any Parameter	
	HR + SV	HR + SVI
Accuracy	90%	90%
MAE	0.431	0.350
RMSE	0.625	0.400
R^2^	0.363	0.442
r Value	0.5544	0.8218
*p* Value	0.0963	0.0035

## Data Availability

The data that support the findings of this study are not publicly available due to confidentiality agreements, ethical restrictions, and institutional review board (IRB) regulations. These restrictions are implemented to protect participant privacy and ensure compliance with institutional policies and applicable legal requirements. The dataset contains sensitive personal information, and the study participants did not provide consent for public data sharing. Therefore, the data cannot be publicly disclosed.
